# Pharmacokinetic Modeling of Ceftiofur Sodium Using Non-linear Mixed-Effects in Healthy Beagle Dogs

**DOI:** 10.3389/fvets.2019.00363

**Published:** 2019-10-17

**Authors:** Jianzhong Wang, Benjamin K. Schneider, Jiao Xue, Pan Sun, Jicheng Qiu, Jonathan P. Mochel, Xingyuan Cao

**Affiliations:** ^1^Department of Veterinary Pharmacology and Toxicology, College of Veterinary Medicine, China Agricultural University, Beijing, China; ^2^Laboratory of Quality & Safety Risk Assessment for Animal Products on Chemical Hazards (Beijing), Ministry of Agriculture and Rural Affairs, Beijing, China; ^3^Biomedical Sciences, SMART Pharmacology, College of Veterinary Medicine, Iowa State University, Ames, IA, United States; ^4^Laboratory of Detection for Veterinary Drug Residues and Illegal Additives, Ministry of Agriculture and Rural Affairs, Beijing, China

**Keywords:** ceftiofur sodium, pharmacokinetics, NLME, monte carlo simulations, dogs

## Abstract

Ceftiofur (CEF) sodium is a third-generation broad-spectrum cephalosporin commonly used in an extra-label manner in dogs for the treatment of respiratory and urinary system infections. To contribute to the literature supporting CEF use in companion animals, we have developed a compartmental, non-linear mixed-effects (NLME) model of CEF pharmacokinetics in dogs (PK). We then used the mathematical model to predict (via Monte Carlo simulation) the duration of time for which plasma concentrations of CEF and its pharmacologically active metabolites remained above minimum inhibitory concentrations (respiratory tract *Escherichia coli* spp.). Twelve healthy beagle dogs were administered either 2.2 mg/kg ceftiofur-sodium (CEF-Na) intravenously (I.V) or 2.2 mg/kg CEF-Na subcutaneously (S.C). Plasma samples were collected over a period of 72 h post-administration. To produce a measurement of total CEF, both CEF and CEF metabolites were derivatized into desfuroylceftiofur acetamide (DCA) before analysis by UPLC-MS/MS. No adverse effects were reported after I.V or S.C dosing. The NLME PK models were parameterized using the stochastic approximation expectation maximization algorithm as implemented in Monolix 2018R2. A two-compartment mamillary model with first-order elimination and first-order S.C absorption best described the available kinetic data. Final parameter estimates indicate that CEF has a low systemic clearance (0.25 L/h/kg) associated with a low global extraction ratio *E* = 0.02) and a moderate volume of distribution (2.97 L/kg) in dogs. The absolute bioavailability after S.C administration was high (93.7%). Gender was determined to be a significant covariate in explaining the variability of S.C absorption. Our simulations predicted that a dose of 2.2 mg/kg CEF-Na S.C would produce median plasma concentrations of CEF of at least 0.5 μg/mL (MIC_50_) for ~30 h.

## Introduction

Ceftiofur sodium (CEF-Na) is a third-generation broad-spectrum cephalosporin (β-lactam antibiotic) which is effective against Gram-positive, Gram-negative, anaerobic, and β-lactamase producing bacteria (1). CEF has been developed and approved for treating bacterial lung diseases in cattle (2), swine (3), and in horses (4). The pharmacokinetics (PK) of CEF has previously been described in cattle (5–8), camels (9), goats (10), horses (11), sheep (12), swine (1), alpacas (13), and rabbits (14).

The metabolism of ceftiofur is similar in rats (15), cattle (15), swine (16), horses (17), and dogs (18) and is characterized by rapid cleavage of the thioester bond to the active metabolite desfuroylceftiofur (DFC) and furoic acid after parenteral administration. Free DFC (which contains an intact β-lactam ring) is the primary microbiologically active metabolite of ceftiofur (6). It is further metabolized to disulfides and also bound to macromolecules in plasma and tissues which are DFC-glutathione disulfide, DFC-cysteine disulfide, 3,3-DFC-disulfide (DFC-dimer), and DFC-protein (19).

The PK of subcutaneous (S.C) CEF crystalline-free acid S.C (20) as well as the PK of CEF-Na S.C (18) have been previously reported in dogs. However, no detailed description of CEF-Na disposition kinetics after intravenous (I.V) dosing is currently available in dogs, which prevents a rigorous assessment of absolute bioavailability in this species. And, despite common off-label use of CEF-Na in veterinary clinics for canine respiratory disease, no formulation is currently approved for use in dogs. In-depth knowledge of the time-course of systemic CEF-Na concentrations will aid in the development of effective CEF-Na formulations for the treatment of canine respiratory and urinary system infections.

The primary aim of this study was to develop a PK model of CEF disposition kinetics in healthy dogs after CEF-Na I.V and CEF-Na S.C dosing. To produce data for model building, we administered either 2.2 mg/kg CEF-Na I.V or 2.2 mg/kg CEF-Na S.C to 12 healthy beagle dogs on two separate occasions. Non-linear mixed-effects (NLME) modeling was used for data analysis, to allow for simultaneous modeling of the I.V and S.C route. Another advantage of NLME modeling lies in the concurrent estimation of between-subject variability, within-subject (i.e., inter-occasion) variability, and individual covariate effects on drug pharmacokinetics (21–23). After model building and validation, the resulting fit was then used to predict (via Monte Carlo simulations) the duration of time for which plasma concentrations of CEF and its pharmacologically active metabolites remained above minimum inhibitory concentrations (MIC50, MIC90) for respiratory tract *Escherichia coli* spp.—the most common respiratory and urinary tract pathogens in dogs. *Bordetella bronchiseptica* and *E. coli* spp. are two of the most commonly reported pathogens in the respiratory and urinary tract of dogs according to previously published studies (20). In addition, ceftiofur is frequently prescribed off-label for infections of the respiratory and urinary tracts in dogs (20).

## Materials and Methods

### Drug Supply and Animals

The commercially available CEF-Na (Sterile Powder, 1 g; Lot No 1708004.2) used in this study was supplied by Qilu Animal Health Products Co., Ltd (Shandong, China). The CEF-Na powder was solubilized for injection by reconstituting the powder in 20 mL of bacteriostatic water for injection to each 1 g vial. Six male and 6 female healthy beagle dogs were included in the study design. Animals ages ranged between 1.5 and 2.5 years old, while dogs weighed between 9 and 12 kg. Dogs were acclimated to the experimental facilities for a minimum of 2 weeks before the start of the study. Dogs were housed individually in solid-floored pens lined with hardwood chip bedding. The animals were individually identified through a combination of cage label, sex, and a permanent ear tattoo. They were fed with a commercial standard feed (Medium-25, Royal Canin, Shanghai, China) and had free access to fresh water. Suitability for inclusion by the study veterinarian was evaluated by physical examination combined with measurement of hematology, clinical chemistry, and coagulation time parameters. General health observations were performed at least daily. The study protocols and experimental design were reviewed and approved by the Animal Use and Care Administrative Advisory Committee of the China Agricultural University (Beijing, PR China).

### Drug Administration and Sample Collection

Dogs were randomly assigned to one of two dosing groups and received either 2.2 mg/kg CEF-Na I.V (cephalic vein) or 2.2 mg/kg CEF-Na S.C (behind the shoulders)—using a block design on sex to ensure that 3 males and 3 females were assigned to each study group. Approximately 2 mL of blood were collected from preplaced cephalic vein catheters or by venipuncture collected directly into heparinized tubes at 0, 0.08 (I.V group only), 0.25, 0.5, 0.75, 1, 1.5, 2, 3, 4, 6, 8, 12, 24, 36, 48, and 72 h post drug administration. The samples were then centrifuged at 2,280 g for 10 min. Plasma samples were then stored at −20°C before further analysis.

### Analytical Methods

Ceftiofur Standards (purity ≥99%, HPLC grade) was supplied from Sigma–Aldrich (St. Louis, MO, USA). All other reagents and materials were analytical grade and supplied from Beijing Chemical Reagent Co., (Beijing, China). Ceftiofur and desfuroylceftiofur metabolites in plasma samples and standards were derivatized to desfuroylceftiofur acetamide (DCA) before analysis by UPLC-MS/MS. This protocol is a modification of existing standard operating procedures for CEF quantification adapted to canine samples (6). In this assay, dithioerythritol is used to convert ceftiofur and all desfuroylceftiofur metabolites containing an intact β-lactam ring to desfuroylceftiofur. Desfuroylceftiofur was then stabilized by derivatization with iodoacetamide to DCA and total CEF equivalent concentration (measured as DCA) was then quantified by UPLC-MS/MS (18). Briefly, the method uses dithioerythritol to cleave any macromolecule bound to desfuroylceftiofur in the serum. The sample was derivatized with iodoacetamide to produce desfuroylceftiofur acetamide. After derivatization, further cleanup was carried out on an Oasis HLB (hydrophilic-lipophilic balance) cartridge (3 cc, 60 mg). The eluate was then collected and evaporated using nitrogen gas. Afterward, the residues were finally dissolved in an aqueous acetonitrile solution. The supernatants were collected and filtrated through a 0.22-μm microbore cellulose membrane and analyzed through UPLC-MS/MS. The UPLC-MS/MS was a Water Quattro Premier. Separation of the compound was accomplished with a Phenomenex column (Kinetex 50 × 2.1 mm i.d. particle size = 2.6 μm, C18, 100 Å). The lower limit of quantification (LLOQ) for the analysis was set at 100 ng/mL. The calibration curves were in good linearity (R2 > 0.998) and ranged from 100 to 5,000 ng/mL. The inter-day and intra-day coefficients of variation—using 200, 1,000, and 4,000 ng/mL standards—were all below 7.58%, while the mean recoveries ranged from 82.15 to 119.44%. All analyses complied with established guidelines on bioanalytical method validation (24).

### NLME Model Building and Evaluation

No outliers were identified after initial data exploration in Monolix datxplore (2018R2, Lixoft, France), such that all data could be pooled together for model building. CEF plasma concentration time-courses from I.V and S.C dosing were analyzed simultaneously using the stochastic approximation expectation maximization algorithm as implemented in Monolix 2018R2 (Lixoft, France). Individual model parameters were obtained by using the full posterior of the conditional distribution. NLME models were written as described by Sheiner and Ludden (25, 26):

$$\begin{array}{lll} y_{ij} & = & F\left(\phi_{i},t_{ij}\right)+G\left(\phi_{i},t_{ij},\beta \right)\cdot \varepsilon_{ij}\\ \phi_{i} & = & \mu \cdot e^{\eta i}\\ \; & \; & j\;\in \left\{1,\ldots ,n_{i}\right\},i\;\in \left\{1,\ldots ,N\right\}, \end{array}$$

Where ***y***_***ij***_ is the observed concentration of CEF equivalent collected from individual ***i*** (of ***N*** total individuals) at time ***t***_***ij***_, and ***j*** indexes the individual sample times from 1 to ***n***_***i***_. ***F***(*ϕ*_***i***_**,*t***_***ij***_) is the predicted concentration of CEF at time ***t***_***ij***_ dependent on *ϕ*_***i***_, the vector of individual parameters (e.g., volume of distribution, clearance). ***G***(*ϕ*_***i***_**,*t***_***ij***_, *β*)·*ε*_***ij***_ is the residual error function of ***F***(*ϕ*_***i***_**,*t***_***ij***_) where *ε*_***ij***_ is an independent random variable distributed in a standard normal distribution i.e., *ε*_***ij***_ **~ *N(0, 1)***. Each individual parameter *θ*_***i***_∈*ϕ*_***i***_ was modeled as a combination of the population mean *μ* (i.e., *θ*_***pop***_) and log-normally distributed error *η*_***i***_ i.e., log(*θ*)~ ***N***(***log*** (*μ*) ~*η*_*i*_).

Convergence of the SAEM algorithm was evaluated by inspection of the stability of the fixed- and random-effect parameters search as well as the precision of parameter estimates—defined via their relative standard error (RSE). Standard goodness-of-fit diagnostic plots, including individual predictions vs. observations, individual weighted residuals (IWRES), and predictions distribution were used to assess the performances of the candidate models. Normality of the random effects was assessed using the Shapiro–Wilk test as well as inspection of the full posterior distribution of random effects and residuals. Selection criteria between competing structural models included the Bayesian information criteria (BIC) and the precision of the model parameter estimates. The BIC was selected over the Akaike Information Criterion (AIC) as it tends to favor more parsimonious models (27).

### Handling of Below Limit of Quantification (BLQ) Data

Data below the LLOQ were modeled by adding to the likelihood function a term describing the probability that the true observation lies between zero and the LLOQ. This LLOQ corrected likelihood function is equivalent to the M4 method as implemented in the most recent release of NONMEM (Version 7.4; ICON Development Solutions).

### Random Effects Correlation Estimates

Visual inspection of the scatterplot of random effects as well as Pearson correlation tests were used to evaluate correlations between model parameters. *P* < 0.05 were considered as statistically significant. In agreement with previous literature (26, 28, 29), several samples of the posterior distribution obtained during the last iteration of the SAEM algorithm, rather than the empirical Bayes estimate (EBE), were used when producing the scatterplot to better assess correlation between model parameters.

### Inclusion of Covariate Relationships

The effect of two continuous covariates (BW and age) and one categorical covariate (sex) were evaluated using the automated Pearson's correlation test and the ANOVA method as implemented in Monolix 2018R2. *P* < 0.05 was used as threshold for statistical significance i.e., for inclusion of a covariate effect in the final NLME model. Age and BW were normalized by their median value and log-transformed during the covariate search.

### Monte Carlo Simulations

After model selection and fit, we used two sets of Monte Carlo simulations to answer two questions. First, we wanted to use our model to visualize the entire distribution of predicted CEF-Na concentration time-courses in dogs, after a dog is administered 2.2 mg/kg S.C. Plotting this prediction distribution against our observations gave us additional insight into the quality of our model predictive ability.

The time period for which CEF plasma concentrations remained effective was defined as the time period for which median CEF plasma concentrations remained above the MIC_50_ (0.5 μg/mL) and MIC_90_ (8 μg/mL) for respiratory tract or urinary tract *Escherichia coli* spp. This time period for which concentration remained above MIC_50/90_ was given the variable name τ_50/90._ MIC values were obtained from previously published canine studies (20). The R 3.4.4 package Simulx 3.3.0 (Monolix 2018R2) was used to simulate CEF plasma disposition kinetic profiles from final Monolix run files.

In the first set of simulations, we simulated a population of 500 females and 500 females and virtually administered CEF-Na at 2.2 mg/kg S.C. Furthermore, using simulation set 1, we were able to explore the probability that—for a S.C dose of 2.2 mg/kg, and a dosing interval of 24 h—the concentration of ceftiofur would remain above a set of predefined plasma concentrations for a given period of time, referred as the pharmacodynamic target (PDT) (%T > MIC = {40, 60, 80, 100}). This approach follows recommendations from the European Committee on Antimicrobial Susceptibility Testing (EUCAST) working group as outlined in Mouton et al. (30). Then, we used the PK data from this simulation to produce prediction distributions of CEF between 0 and 40 h.

In the second set of simulations, we simulated the median CEF PK of male and female dogs after S.C dosing with 1–5 mg/kg (in steps of 0.1 mg/kg) of CEF-Na. Using this second simulation set, we were able to calculate the median τ50 and median τ90 for both males and females as a function of CEF-Na dosage.

## Results

### Animals

No noticeable signs of discomfort were observed upon injection of CEF-Na and no complications resulted from CEF exposure.

### Pharmacokinetic Model

**Figure 1:**
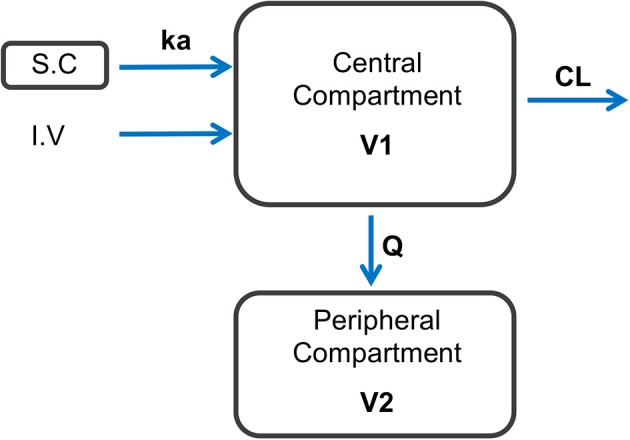
Schematic representation of the final model structure used to represent the dynamics of CEF following I.V and S.C dosing in healthy beagle dogs. A two-compartment pharmacokinetic model with first-order elimination and first-order absorption after S.C dosing with CEF best fitted the observed data. ka: 1-st order absorption rate following S.C dosing with CEF; CL, CEF systemic clearance; Q, inter-compartmental clearance; V1, central volume of distribution; V2, peripheral volume of distribution.

**Figure 2:**
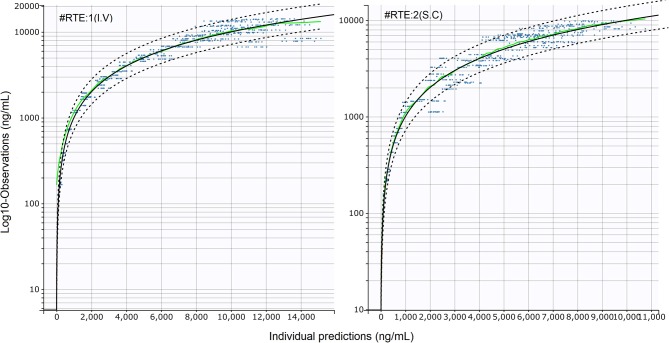
Standard goodness-of-fit (sGOF) diagnostics: individual predictions vs. observations (log scale). **Left**: I.V (#RTE: 1); **Right**: S.C (#RTE: 2). The robustness of fit and predictive performances of the final model were supported by the inspection of the sGOF plots. Blue dots: observations; green line, identity line; dotted black lines: 90% prediction interval; red dots: censored (i.e., below the quantification limit) data. As described by Nguyen et al. (31), observations were displayed on a log-scale to better evaluate the quality of fit.

**Figure 3:**
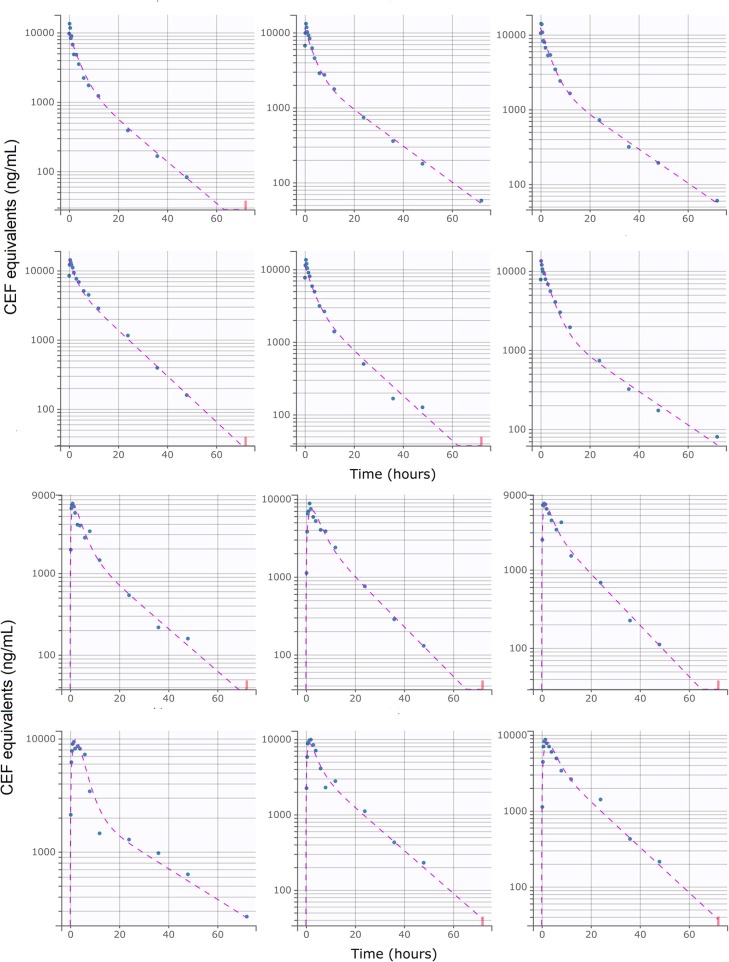
Individual predictions of CEF equivalent plasma concentrations in healthy beagle dogs from the final selected model. **Upper**: I.V (#RTE: 1, *n* = 6); **Lower**: S.C (#RTE: 2, *n* = 6). Scatter plot of observed (blue dot) and predicted (dashed purple line) individual concentration vs. time after dosing. The full model was able to describe the individual time-course of CEF equivalents for all administration schedules with excellent accuracy, as shown by the quality of the individual fits. Below LLOQ data are represented with red dots.

A total of 198 plasma concentrations of CEF and metabolites (measured as DCA by UPLC-MS/MS) from both I.V and S.C dosing groups were pooled together and simultaneously modeled using NLME. Only 4.0 % (8/198) data were found to be below the LLOQ of the UPLC-MS/MS validated method. A two-compartment mammillary PK model with first-order elimination and first-order absorption for the S.C route, was found to best fit the pharmacokinetics of CEF equivalents in plasma based on standard goodness-of-fit diagnostic plots, precision of parameter estimates (RSE), as well as comparison of BIC between competing structural models (Figures 1–3) (31). A log-normal error model best captured the residual variability in the model (Supplemental Figure 1A). Individual effects were approximately log-normally distributed around their respective population mode (Supplemental Figure 1B). After inspection of the correlation matrix of the random effects (Supplemental Figure 1C), a correlation between CEF systemic clearance (*Cl*) and central volume of distribution (*V*_1_) was identified and subsequently included in the structure of the statistical model (*corr*(*V*_1_, *Cl*) ≅. 999, *P* ≤ 0.01). Results from the automated covariate search as implemented in Monolix 2018R2 identified sex as a significant covariate on CEF subcutaneous absorption rate (P ≤ 0.01). Gender was therefore included in the final model structure, using the following relationship:

$$\begin{array}{l} log\left(ka_{i}\right)=log\left(ka_{pop}\right)+\beta \cdot sex_{i=f}\;+\;\eta_{i} \end{array}$$

Where *sex*_*i* = *f*_ is equal to 1 if individual *i* is a female and 0 otherwise. *ka*_*pop*_ is the population subcutaneous absorption rate for male dogs and β is the effect of the categorical covariate (i.e., sex) on *k*_*a*_. Using final parameter estimates from the model, CEF absorption rate was estimated to be two times greater in male vs. female dogs.

### Parameters Estimates

Final parameter estimates and relevant RSEs are tabulated in Table 1. The precision of the final estimates was high (RSE ≤ 15%), reflecting an accurate and stable parameterization of the model. The total systemic clearance of CEF was estimated to be low 0.25 L/kg/h (32), with an estimated volume of distribution of 2.97 L/kg (1.69 and 1.28 L/kg for the volume of the central and the peripheral compartment, respectively).

**Table 1:** Estimated model parameters and their associated inter-individual and inter-occasion variability for CEF pharmacokinetics in dogs.

**Parameter**	**Symbol**	**Unit**	**Point estimate**	**RSE (error %)**	**IIV (%)**
Clearance	CL	L/h/kg	0.25	8.29	24
Absorption (S.C)	Ka	1/h	1.43	11.9	–
Central compartment volume of distribution	V1	L/kg	1.69	6.9	32.4
Peripheral compartment volume of distribution	V2	L/kg	1.28	12.9	25.7
Inter-compartmental clearance	Q	L/h/kg	0.16	13.6	–
Bioavailability (S.C)	F	%	93.7	11.4	52
Correlation (*CL* and *V1*)	corr(cl_v1)	%	99.9	6.24	–
Coefficient (Ka and sex)	β_*sex*_	–	−0.643	20.1	–

*IIV, Inter-Individual Variability, expressed as CV%; S.C, Subcutaneous; RSE, Relative Standard Error, –, Model parameter estimated to converge to a null value and fixed to 0. More details on the abbreviated parameters can be found in the legend of Figure 1*.

Cardiac output, *Q*, was approximated using the formula, *Q*≅180 × *BW*^−0.19^ (32). The global extraction ratio of CEF (*E* = *Cl*/*Q*) was estimated to be low (*E* = 0.02). The absolute bioavailability of CEF was estimated as 93.7%.

### Model Predictions

**Figure 4:**
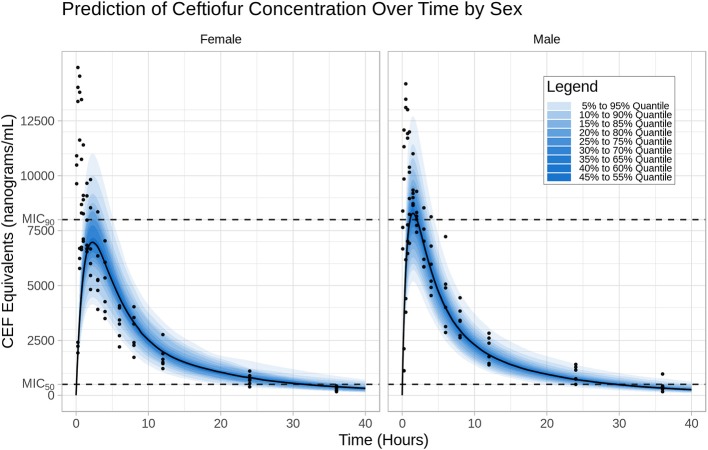
Prediction distribution of CEF pharmacokinetics. **Left**: I.V (#RTE: 1); **Right**: S.C (#RTE: 2). The theoretical distribution of CEF PK was produced by 500 Monte Carlo simulations from the final model. Briefly, the experiment was replicated virtually 500 times, allowing for each quantile (from 5 to 95 in steps of 5 i.e., {5,10,15,…,90,95}) to be estimated 500 times. The blue areas are ranges of quantiles and the blue points are observations for comparison.

The prediction distribution of CEF equivalents over time after 2.2 mg/kg CEF-Na S.C administration suggests that CEF total concentrations (measured as DCA) would remain below the MIC_90_ concentration threshold (8 μg/mL) for most of the dosing interval, except for individuals in the upper percentiles of the simulated population (Figure 4). Also, because male dogs had a higher estimated CEF absorption rate than females, their peak exposure (i.e., C_max_) was predicted to be greater than in female dogs.

**Figure 5:**
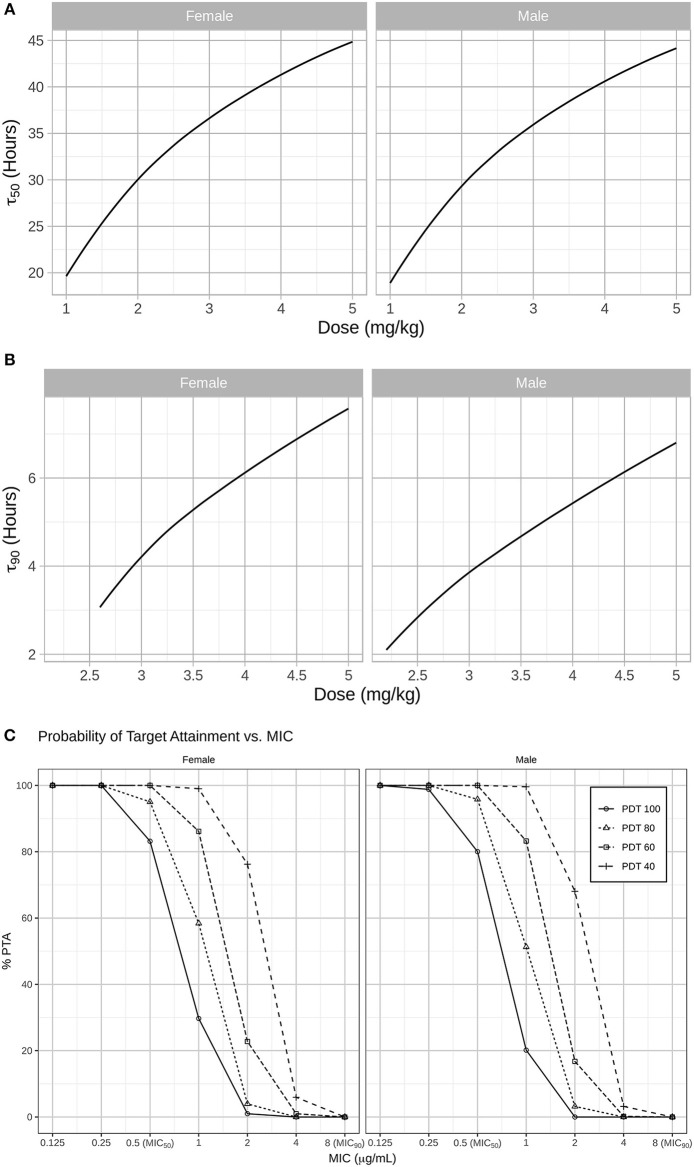
**(A)** In a second step, simulations were used to predict for how long CEF plasma concentrations remained above the MIC_50_ (0.5 μg/mL) and MIC_90_ (8 μg/mL) for *Respiratory tract Escherichia coli* spp. in both males and females dogs after administration CEF-Na at 2.2 mg/kg S.C. Specifically, the median PK of males and females after S.C dosing with 1 to 5 mg/kg of CEF-Na was simulated to derive the median **τ**_50_ (**B**; left panel: male; right panel: female) and median τ_90_ (**C**; left panel: male; right panel: female) as a function of CEF-Na dosage. **(C)** Probability of Target Attainment (PTA%) for various pharmacodynamic targets (40, 60, 80, 100) and a range of MIC values. As suggested in **(B)**, at an MIC of 0.5 μg/mL (MIC_50_), nearly 100% of the target population is expected to reach the pharmacodynamic target. This percentage drops as the MIC increases.

Results from our model-based simulations suggest that after one dose of 2.5 mg/kg CEF-Na S.C, ceftiofur concentrations would remain above the MIC_50_ threshold (0.5 μg/mL) for almost 1.5 days in both male and female dogs (Figure 5A). We also found that ceftiofur peaks at a higher plasma concentration in males, but the probability that ceftiofur plasma concentrations remain above various target MICs for target dosing intervals is higher in females than it is in males. We found that even at relatively high target MICs (1.0 μg/mL), both female and male dogs remain above those targets for reasonable periods of time (~9.6 h, hence 40% of the dosing interval) with high probability (99% of simulated females, and 100% of simulated males). As the target time periods increases to 24 h (PDT = 100%), we see a steep decrease in probability of remaining over MICs above 0.5 μg/mL. More precisely, 30% of simulated females and 20% of simulated males remained above 1.0 μg/mL for at least 24 h (see Figure 5C and Table 2 for further details).

**Table 2:** Probability of Target Attainment (PTA%) for various pharmacodynamic targets following daily dosing with ceftiofur (2.2 mg/kg, S.C).

	**MIC (μg/mL)**	**40%**	**60%**	**80%**	**100%**
**Fraction of First 24 h Post Administration** **>** **MIC**					
Females	0.125	100	100	100	100
	0.25	100	100	100	100
	0.5	100	100	95	83
	1	99	86	58	30
	2	76	23	4	1
	4	6	1	0	0
	8	0	0	0	0
Males	0.125	100	100	100	100
	0.25	100	100	100	99
	0.5	100	100	96	80
	1	100	83	51	20
	2	68	17	3	0
	4	3	0.2	0	0
	8	0	0	0	0

*In these tables, the probability of the plasma concentration of ceftiofur remaining above the potential MIC for the percentage fraction of dosing interval is displayed. In general, the predicted ceftiofur concentration in female dogs remained above target concentrations longer than for male dogs*.

In contrast, our predictions of median τ_90_ as a function of dosage indicate that even when administered at unrealistically high doses of CEF-Na S.C (~5 mg/kg), CEF concentrations would remain above MIC_90_ levels for no more than 8 h (Figure 5B).

## Discussion

Since 1991, Ceftiofur has been approved and extensively used by veterinarians in the treatment of bacterial infections in cattle, swine, and horses. This study constitutes the very first pharmacokinetic report of CEF-Na absolute bioavailability in dogs, allowing for the proper estimation of CEF systemic clearance and volume of distribution (as opposed to *apparent* clearance and distribution volume estimated with extravascular dosing of CEF-Na). Previously, the PK of ceftiofur in dogs has only been described in two studies. First, the PK of a single subcutaneous dose of ceftiofur crystalline-free acid has been described using non-compartmental analysis (20). Second, the PK of CEF-Na S.C has been reported using non-linear least squares regression (18). Results from our analysis suggest that the absolute bioavailability of CEF-Na S.C is higher in dogs than in cattle (61.12%) (6). We observed an apparent systemic clearance (CL/F) for dogs (0.12 L/h/kg) that is higher than previously reported in dogs (0.039 L/h/kg) (20) and lower than previously reported in cows (0.26 L/h/kg) (6). Lastly, we estimated a lower S.C absorption rate (1.43 1/h) than previous estimates of CEF-Na S.C absorption in dogs (2.26 1/h) (18).

In our analysis, CEF and desfuroylceftiofur metabolites (containing an intact β-lactam ring) in plasma samples were derivatized to DCA (18), and total CEF equivalent concentrations (measured as DCA) were quantified by UPLC-MS/MS. Free concentrations only accounts for about 10% of total CEF equivalents (6). However, protein binding of desfuroylceftiofur is known to be reversible, such as protein-bound desfuroylceftiofur acts as a reservoir for release of active therapeutic drug at the site of infection (33). Hence, measurement of DCA regardless of protein binding was used for simulation of what-if scenarios and dose optimization in our experiment.

NLME models are a versatile statistical tool for quantifying variability in drug disposition as a function of individual patient characteristics (i.e., covariates, such as age, sex, and bodyweight) (34–36). NLME modeling also enables decoupling of intra-individual variability, inter-individual variability, and residual error. This allows to individually consider the many factors that could affect drug exposure in any given individual. Pooling data from I.V and S.C dosing with CEF (totaling 198 concentrations), the disposition kinetics of CEF equivalents was best modeled using a two-compartmental mammillary model with first-order elimination and first-order absorption from the S.C injection site. Our final selected model precisely captured the individual PK of total CEF equivalents over time in both dosing groups. Results from the automated covariate analysis in Monolix 2018R2 further suggest that sex has a significant effect (β_*sex*_ = −0.643 ± 20.1%) on CEF absorption rate following subcutaneous administration. This was also supported by the inspection of the distribution of the estimated individual absorption parameters (i.e., ka_i_). Specifically, CEF absorption rate was estimated to be two times greater in male vs. female dogs, and our model-based simulations confirmed the potential need for dose adjustment based on sex in dogs. To the best of our knowledge, no previous studies had reported an effect of sex on ceftiofur PK in dogs or any other species.

Importantly, using final parameters estimates from the NLME model, we could simulate “*what-if* ” scenarios to evaluate various dosing schedules for CEF-Na S.C in dogs. The most important risk factor for emergence of resistance is repeated exposure of bacteria to suboptimal concentrations of antimicrobials related to the selection of inappropriate dosing schedules (36). As a cephalosporin antibiotic, CEF exhibits time-dependent bactericidal activity i.e., plasma concentrations of CEF must be maintained over relevant MIC levels for an extended period of time. As such, the amount of time that CEF concentrations remain above the MIC_XX_ (i.e., τ_xx_) is the PK-PD best index for predicting drug efficacy (37).

According to previous research with cephalosporins, τ_xx_ should be at least 50% (and preferably ≥ 80%) of the dosage interval to achieve optimal bactericidal effect without inducing resistance (38). Using the EUCAST approach outlined by Mouton et al. (30), we predict that for MICs of ~1.0 μg/mL we achieve a pharmacodynamic target of 80% with relatively high probability (~ 60%) for once daily dosing at 2.2 mg/kg S.C. For smaller MICs (≤0.5 μg/mL), we achieve that target with high probability (>90%). Probability of target attainment drops off steeply for high MIC values. In our simulations, doubling the daily dose of ceftiofur (e.g., from 2.2 to 4.4 mg/kg) produces ~1.5 times greater time above MIC_50_ for *E. coli* spp.

In summary, our simulations suggest a wide spectrum of viable dosing regimens and dosages for CEF-Na subcutaneous in dogs. However, producing a definitive recommendation of dosing interval for CEF in dogs was not within the primary scope of this study. As such, further studies in client-owned animals with clinical disease are required to validate and build on our preliminary findings in healthy dogs.

## Limitations

Our study had several limitations. First, this experiment was performed in healthy dogs and model-based predictions of CEF disposition kinetics may not extend to dogs with bacterial infection, impaired hepatic function, or impaired renal function. Second, we chose to refer to MIC values from previous studies rather than culturing clinical pathogens as a part of the sampling process. Third, we have no information about the free concentration fraction of CEF in plasma. Finally, and with respect to our experimental design, this study solely reports the disposition kinetics of CEF after a single dose of CEF-Na, with no information about systemic accumulation and steady-state pharmacokinetics of CEF in dogs.

## Data Availability Statement

The datasets generated for this study are available on request to the corresponding author.

## Ethics Statement

The animal study was reviewed and approved by Animal Use and Care Administrative Advisory Committee of the China Agricultural University.

## Author Contributions

XC and JW were involved in the original design and execution of the study. PS, JX, JW, and JQ were responsible for the animal experiments. BS, JW, and JM performed the NLME data analysis and wrote the manuscript. All authors have read and approved the final version of the manuscript. All authors contributed to the preparation of the manuscript.

### Conflict of Interest

The authors declare that the research was conducted in the absence of any commercial or financial relationships that could be construed as a potential conflict of interest.

## Supplementary Material

Supplemental Figure 1 **(A)** Scatter plots of model residuals (Individual Weighted Residual, IWRES) vs. predicted CEF concentrations. Brown line: regression curve; red dots: censored (i.e., below the quantification limit) data. A suitable model should have the following features: (i) residuals (IWRES) should be centered around a mean value of 0 (i.e., the regression curve should mirror a horizontal line of *y* = 0), (ii) a homogeneous distribution about the mean. **(B)** Boxplot of the distribution of the random effects (η_*i*_). Box: 1st quartile, median and 3rd quartile; Whiskers: ±1.5 interquartile range; Red crosses: statistical outliers. The normality of the random effects was further confirmed by the homogeneous distribution of the random effects (η_*i*_) around a mean value of 0. **(C)** Correlation matrix of the random effects (i.e., the ηi). Most correlations were deemed insignificant (coefficient ≤ 0.3, *P* > 0.05), with the exception of the correlation between CEF clearance and volume of distribution (V1): corr_V1_Cl = 0.99 ± 0.05 (*P* < 0.05).
