# Supplementary figures and images for: Pharmacokinetic Modeling of Ceftiofur Sodium Using Non-linear Mixed-Effects in Healthy Beagle Dogs
Source: Front Vet Sci. 2019 Oct 17;6:363. doi: 10.3389/fvets.2019.00363 (PMC6811611; doi:10.3389/fvets.2019.00363)

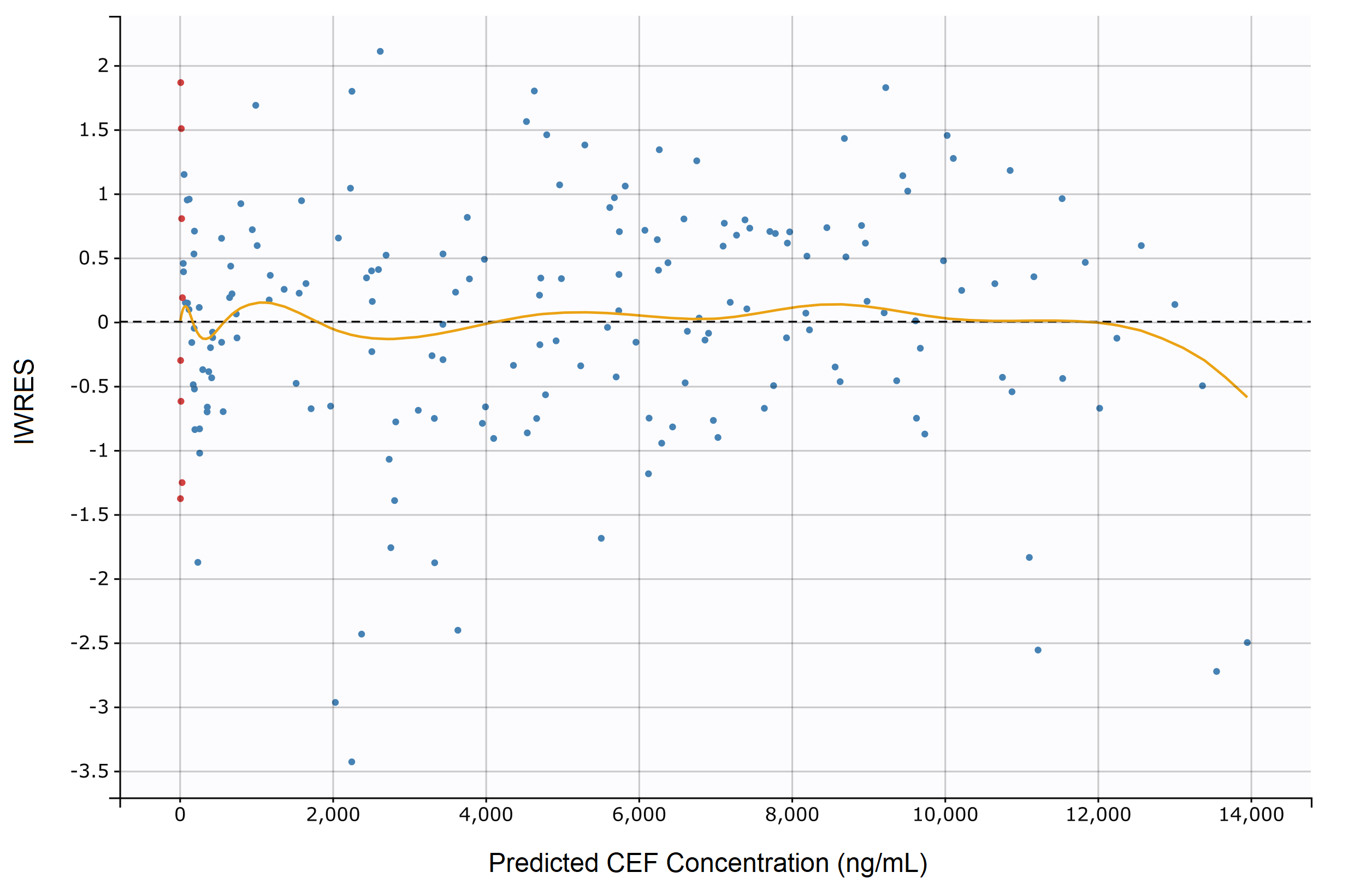

Supplement: Supplemental Figure 1 — (A) Scatter plots of model residuals (Individual Weighted Residual, IWRES) vs. predicted CEF concentrations. Brown line: regression curve; red dots: censored (i.e., below the quantification limit) data. A suitable model should have the following features: (i) residuals (IWRES) should be centered around a mean value of 0 (i.e., the regression curve should mirror a horizontal line of y = 0), (ii) a homogeneous distribution about the mean. (B) Boxplot of the distribution of the random effects (ηi). Box: 1st quartile, median and 3rd quartile; Whiskers: ±1.5 interquartile range; Red crosses: statistical outliers. The normality of the random effects was further confirmed by the homogeneous distribution of the random effects (ηi) around a mean value of 0. (C) Correlation matrix of the random effects (i.e., the ηi). Most correlations were deemed insignificant (coefficient ≤ 0.3, P > 0.05), with the exception of the correlation between CEF clearance and volume of distribution (V1): corr_V1_Cl = 0.99 ± 0.05 (P < 0.05). [file Image_1.TIF]

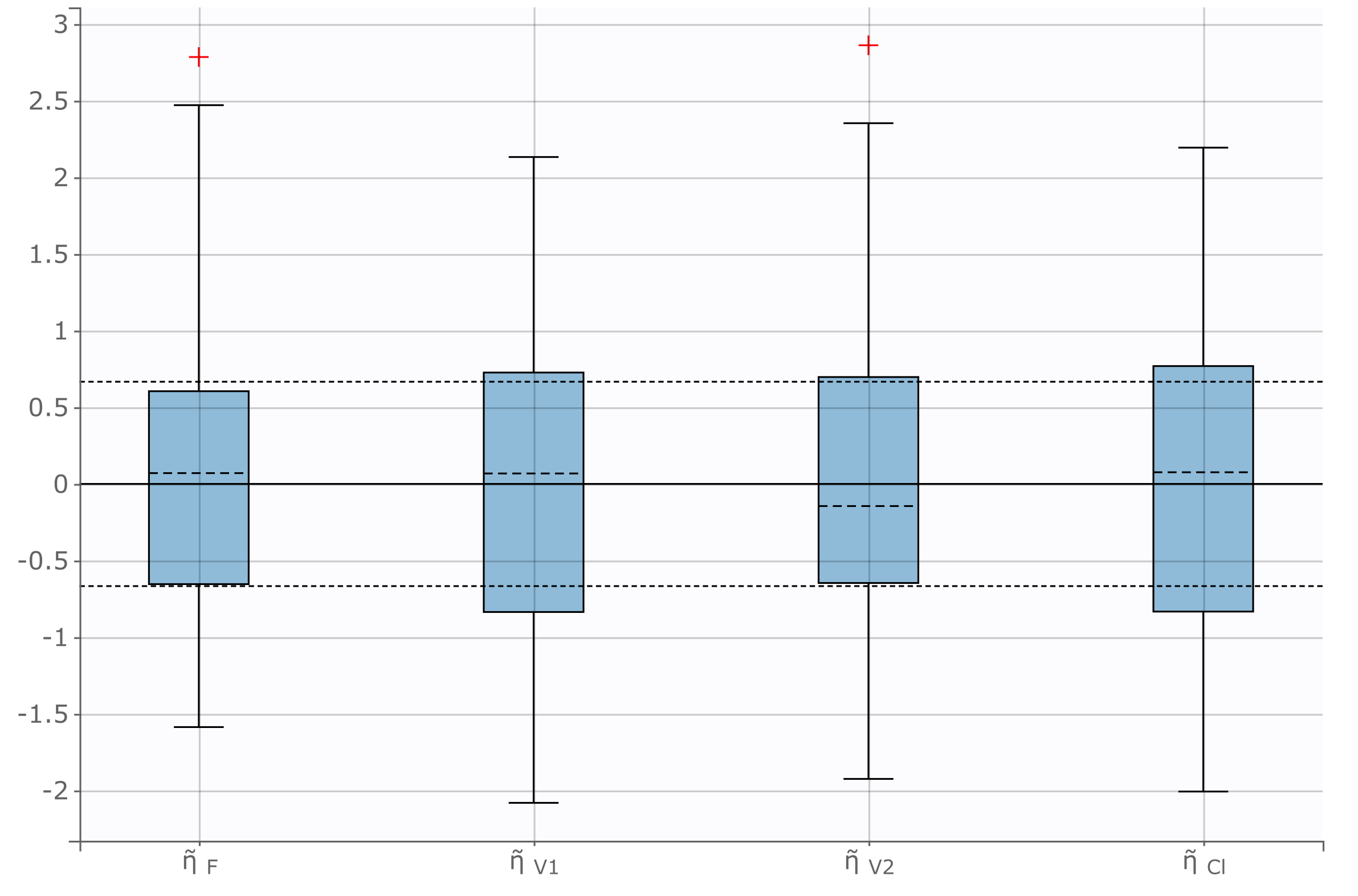

Supplement: Supplementary file 2 [file Image_2.TIF]

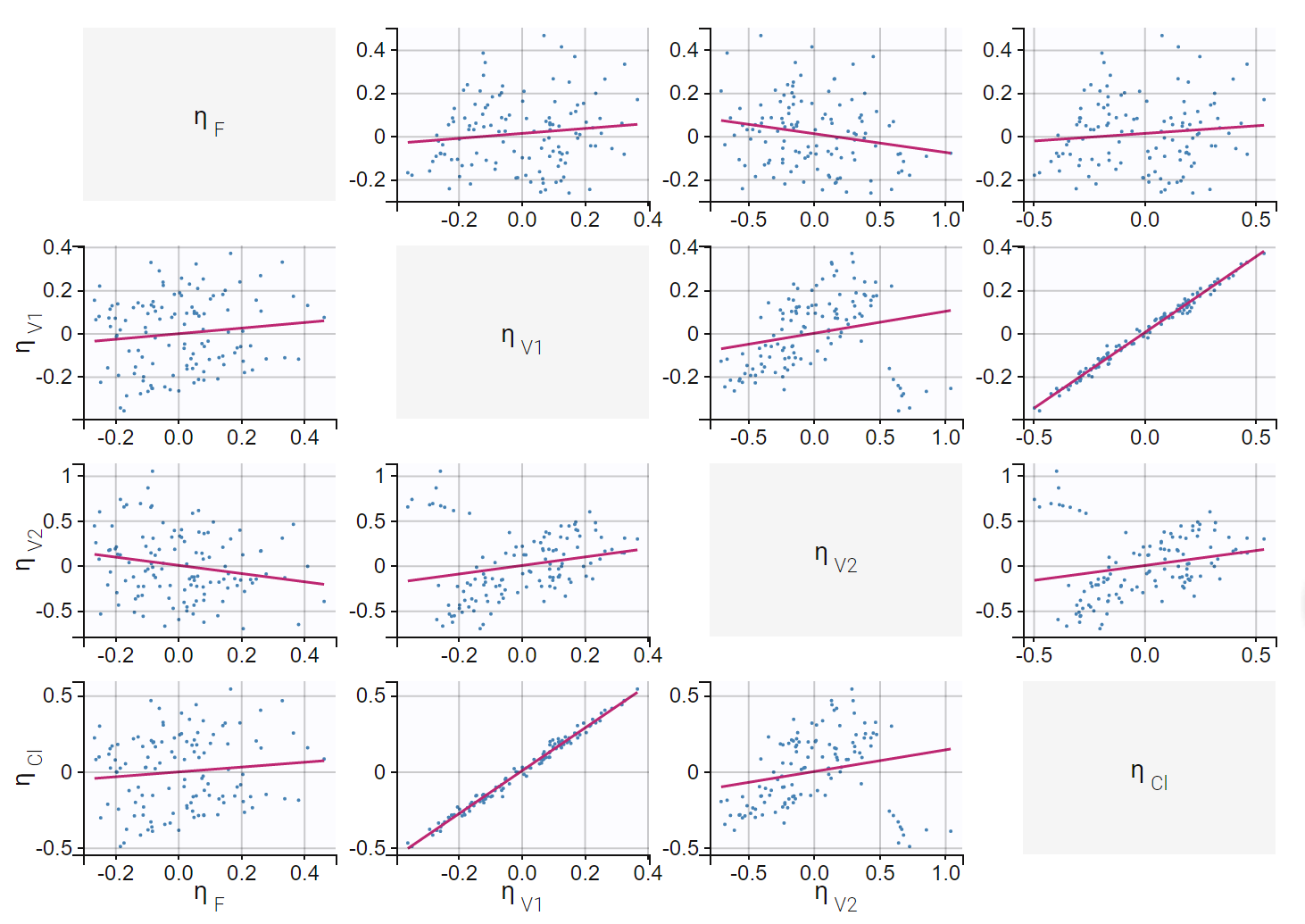

Supplement: Supplementary file 3 [file Image_3.TIF]
